# Payments for Conservation of Animal Genetic Resources in Agriculture: One Size Fits All?

**DOI:** 10.3390/ani11030846

**Published:** 2021-03-17

**Authors:** Luka Juvančič, Renata Slabe-Erker, Marko Ogorevc, Adam G. Drucker, Emil Erjavec, Danijela Bojkovski

**Affiliations:** 1 Biotechnical Faculty, University of Ljubljana, Groblje 3, 1230 Domzale, Slovenia; emil.erjavec@bf.uni-lj.si (E.E.); danijela.bojkovski@bf.uni-lj.si (D.B.); 2 Institute for Economic Research, Kardeljeva Ploscad 17, 1000 Ljubljana, Slovenia; erkerr@ier.si (R.S.-E.); ogorevcm@ier.si (M.O.); 3 Bioversity International, Via dei Tre Denari 472/a, Maccarese, 00054 Rome, Italy; a.drucker@cgiar.org

**Keywords:** animal genetic resources, local breeds, economic valuation, conservation tender, agri-environmental measures

## Abstract

**Simple Summary:**

Maintaining minimum population sizes for local livestock breeds is a key goal in the conservation of animal genetic resources. As markets and livestock production systems have tended to favour a narrow base of high-output improved breeds, countries have had to use financial and other incentives to motivate breeders to keep local breeds. This paper explores the potential for more cost-effective alternatives to the most commonly used financial incentive, a fixed payment per animal or livestock unit. We compare the current fixed payment incentives for local breeds under the Slovenian Rural Development Programmme with those instead determined through a competitive tender approach. A stated preference survey was realised to determine the conditions under which breeders would be willing to participate in such an incentive system based on differentiated payments. Willingness to accept (WTA) payment for conservation was found to differ significantly from actual payment levels, being lower for the local sheep and goat breeds, and higher for the local pig breed. This suggests that implementation of differentiated payments would be more cost-effective; particularly when accompanied by measures to streamline administrative requirements, improve access to breeding stock and target support for local breed market valorisation (e.g., promotion of value chains based on designated quality schemes).

**Abstract:**

Local livestock breeds in Slovenia have been eligible for financial incentives in the form of a fixed payment per livestock unit (LU) since 2002. The scheme has however not been successful in reversing the erosion of animal genetic resources (AnGR). This paper investigates an alternative, whereby incentive payments would better reflect breeders’ actual opportunity costs. The paper contributes to the limited existing body of knowledge related to the use of tender mechanisms in the design of the payments for agrobiodiversity conservation schemes (PACS), particularly for AnGR. Empirical findings draw on the results of a stated preference survey involving 301 farmers in Slovenia, engaging, or being potentially able to engage, in the rearing of local pig, sheep and goat breeds. Interval and logistic regression model results suggest that willingness to accept (WTA) conservation support significantly differs from actual payment levels. The estimated WTA was found to be 27% lower for the local sheep and goat breeds and 5% higher for the local pig breed, suggesting that differentiated incentive payments would provide a more cost-effective alternative. Additional analysis of breeders’ preferences and motives for engaging in local livestock breed production further informs understanding regarding AnGR conservation policy and the importance of accompanying actions to reverse negative population trends. These include reducing administrative barriers and enhancing the market valorisation of local breeds.

## 1. Introduction

In recent decades, concerns regarding the need to conserve genetic resources for food and agriculture in order to render agricultural production systems and rural livelihoods more resilient to shocks and stresses have been growing [1,2]. The loss of diversity and diversified production systems resulting from the intensification of agricultural practices has influenced local food system sustainability [3]. Such impacts relate to the supply of a range of important provisioning, regulatory and cultural ecosystem services, many of which are public goods. These include national food security derived from agroecosystem resilience to pests, diseases and extreme climatic events [2,4,5].

The economic value of such ecosystem services has been shown to be significant [6]. Yet, while the costs of their provision fall locally on the farmer, many of the benefits are regional, national and even global. In the absence of mechanisms to internalise such values, these are likely to be left unaccounted for in farmer production system decisions, leading to the maintenance of less than socially desirable levels of agricultural biodiversity [7]. In recognition of this divergence between the private costs and public benefits of conserving biodiversity in general [8], the Convention on Biological Diversity in the Aichi Target 3 has specifically recognised the need for incentive mechanisms [9]. A wide range of potential policy-driven and voluntary incentive mechanisms exist [10].

Payment for environmental services (PES) is one such type of incentive mechanism that has been widely used for securing public good ecosystem services (see Wunder et al. [11], Salzman et al. [12], and Börner et al. [13] for recent overviews). However, its application in the context of biodiversity has been limited (less than 2% of the PES schemes evaluated by Grima et al. [14]) and that related to agrobiodiversity even more. Examples include EU support payments for threatened livestock breeds under Regulations 1257/99 [15] and 1750/99 [16]; and crop genetic resource-related payments for agrobiodiversity conservation services (PACS) schemes from Latin America, Zambia and India (see Drucker and Ramirez [17], Wainwright et al. [18], and Krishna et al. [19]).

Various challenges associated with agri-environment contract design have been identified. These include information asymmetries, transaction costs, uncertainty over property rights and lack of incentives for entrepreneurship (see Schilizzi and Latacz-Lohmann [20] for a comprehensive review). Many PES schemes based on fixed pricing rules are considered to have overpaid farmers either because of inadequate analysis of supply–demand dynamics as the programmes had income-support objectives in addition to environmental objectives, or because it was administratively too costly to determine farmer-specific payment rates [21,22]. Consequently, conservation auctions (tenders) have been advocated as a mechanism for addressing such challenges [20] and have been applied across a wide range of contexts, including agriculture-related ones (for example, see Stoneham et al. [23], Kirwan et al. [24], Latacz-Lohmann and Schilizzi [25], and Klimek et al. [26]). Application in developing countries have been more limited [27], although the above-mentioned PACS crop-related examples all used such an approach.

Wainwright et al. [18], using a stated preference approach, provide a rare example of a (hypothetical) livestock breed-related conservation tender approach in Romania. They find that while farmer willingness to accept (WTA) was well within the range of the Rural Development Programme (RDP) support payments available, relatively few farmers (8%) were actually likely to qualify for such support. Non-monetary barriers to participation (e.g., minimum field sizes and herd book registration requirements) were found to be significant.

Other stated preference approaches applied in the European context specifically to AnGR have shown that there are high benefit-cost ratios that can be associated with conservation interventions. In Cicia et al. [28], in the context of the wild Pentro horse breed in Italy, it is perhaps not surprising that many of the benefits were associated with its non-market values, despite the fact that horse meat consumption is popular in many parts of Italy. However, even the non-market values associated with threatened breeds raised for more commercial reasons has also been found to be significant. Over 80% of the benefits associated with threatened Alistana-Sanabresa cattle breed conservation in Spain, and Modicana and Maremmana cattle breeds in Italy, were shown to be associated with public good ecosystem service provision, such as that related to landscape maintenance, cultural, option and existence option values. The authors conclude that, consequently, livestock breed conservation strategies should be identified accordingly, with the aim of securing such breed-related functions, as these are the ones that people value most and thus have the highest potential to maximise societal welfare [29,30]. Nevertheless, the existence of direct use (breed-related product) values imply that market valorisation/development of niche product markets and agri-tourism aimed at enhancing the private good values associated with conservation may still form an important element of a conservation and use strategy for threatened breeds. While, Signorello et al. [31] in an EU-wide study also consider that policies consistent with market valorisation have a role to play in addition to direct payments, they note that conservation interventions based on direct payments have failed to prioritise adequately; for example by differentiating between breeds with different extinction probabilities and contributions to overall diversity.

Our paper adds to this body of knowledge by exploring the cost-effectiveness of Slovenian schemes involving fixed payments per animal, which are the most commonly applied incentives for AnGR in Europe [32], relative to one based on differentiated payments.

A stated preference survey based on a (hypothetical) conservation tender is used to investigate the conditions under which farmers would be willing to participate in a Slovenian AnGR conservation programme based on differentiated payments capable of better accounting for differing risk statuses, breed production costs, spatial concentration and market potential. The competitive nature of the tender approach provides a mechanism through which information asymmetries can be overcome by providing farmers with an incentive to reveal their true opportunity costs, while accounting for both their market and non-market preferences.

### Background—Animal Genetic Resources Policy Framework in the EU and Slovenia

The economic and social importance of animal genetic resources conservation and use in agriculture is widely recognised [33,34,35]. Related policy development is of high priority under the EU strategy for Biodiversity [36] and the Farm to Fork strategy [37] under the European Green Deal [38]. The conservation and sustainable use of AnGR requires international and national strategies, as well as specific measures in terms of policy related to direct support, market valorisation of breeds and their products, effective breeding programs and raising people’s awareness regarding the importance of AnGR.

Payment per head of registered breeding animal is the most common form of support for AnGR in European Union (EU) member states [32,39]. These payments typically form part of the Common Agricultural Policy (CAP) agri-environmental schemes. These are implemented through Rural Development Programmes (RDPs) and, post-2023, as part of a newly established CAP Strategic plan [40]. Since their introduction in 1992, agri-environmental schemes have been gaining steadily in terms of their policy importance, as evidenced by the total amount of support available under the European Agricultural Fund for Rural Development (EAFRD) [41].

International trade rules and commitments stipulate that agri-environmental payments should only compensate farmers for the lower yields and/or higher costs of production associated with their participation in agri-environmental schemes [42]. Translated into the current legal framework of the CAP [43], the maximum amount of support payable to the farmers is capped at EUR 200/Livestock Unit (LU). However, documents outlining the CAP post-2023 regulatory framework [44] indicate that the current incentive mechanism is likely to change; partly as a result of a significant reduction in the relevant section of the CAP budget [45] and partly due to the shift of the operating logic of CAP agri-environmental payments, rewarding performance instead of compliance. Policy developers are thus confronted with a challenge to render future agri-environmental payments, including PACS, more result-oriented and cost-effective.

The paper highlights the case of agri-environmental payments for the conservation of animal genetic resources in Slovenia. National legislation recognizes twelve livestock breeds as being native to Slovenia: four sheep and three horse breeds, one breed of cattle, pig, goat and chicken, as well as a subspecies of the western honey bee. As can be inferred from the status and population trends of local livestock breeds (Appendix C) seven breeds are critically endangered, three are endangered, and one is vulnerable. None of the above-mentioned breeds are included in quality assurance or certification schemes, although some food products deriving from these breeds are increasingly valorised through private labels.

The introduction of incentive payments for local livestock breeds coincided with the country’s first RDP 2004–2006 [46]. As stipulated by the provisions of the former legal framework [15], the level of payment was based on the calculation of income foregone due to higher production costs per unit of output associated with the production of local breeds. During the first implementation period defined by the RDP 2004–2006, payments were based on a fixed amount per animal and an average of 12,000 animals were enrolled in the scheme [46]. In the second iteration of the support scheme for local breeds in Slovenia under the RDP 2007–2013 [47], payments were set at EUR 89.38/LU, equivalent to EUR 13.41 for sheep and goats, and EUR 26.81 EUR for pigs, which is lower than during the previous period. Under the 2014–2020 RDP [48], payments have increased to EUR 193.62/LU and are now higher than they were during the first period. While a more detailed comparative review of incentive payments and population trends are presented in Appendix B, Table 1 reports only the levels of incentive payments allocated to the animal species and categories which are dealt with further in our study.

## 2. Materials and Methods

### 2.1. Study Area Description

The Krškopolje pig and Bela Krajina Pramenka sheep originate from the Southeastern lowlands of Slovenia while the Drežnica goat from the mountainous North-West. According to national risk status thresholds, which draw on FAO guidelines [49], the pig breed is considered to be endangered while the sheep and goat breeds are critically endangered. Threat level categorization of breeds is based on three parameters: population (number of breeding females and population trends), inbreeding rate and geographical concentration and four categories of endangerment (listed in descending order of risk): (i) critically endangered; (ii) endangered; (iii) vulnerable; and (iv) not at risk).

The Drežnica goat is dual-purpose (milk and meat production) and the only local goat breed found in Slovenia. The breed shows excellent adaptability to the high mountain pastures and harsh rearing conditions found in the Slovenian Alps. A long history of legal restrictions or outright bans of goat grazing in the area [50] has contributed to the persistently low population of this breed. According to the Register of Breeds [51], the population size in 2003 was 550 animals, increasing to 754 registered breeding animals by 2018 (see Table 2). This number however remains insufficient to result in a change to its critically endangered threat status.

The Bela Krajina Pramenka is one of four Slovenian local sheep breeds. With only 200 breeding animals remaining in 1995, the breed was very close to extinction. Since then, despite remaining critically endangered, population numbers have improved substantially, reaching 1070 by 2018 [51]. Due to its excellent adaptability to poor production environments, (e.g., rocky karst pastures) and good meat quality, the breed is highly appreciated by local farmers for lamb production.

The Krškopolje pig or black-belted pig is the only local Slovenian pig breed. It is adapted to poor production environments and excellent for outdoor rearing. It has a large appetite, high disease resistance, good maternal traits, and moderate fertility traits. The breed is gaining popularity for its ability to produce excellent lard and high-quality meat [52,53]. It is particularly appropriate for the elaboration of traditional cured meat products. Like the Drežnica goat, keeping the Krškopolje pig has been subjected to outright bans, including between 1961–1990 the culling or castrating of boars as part of the national breed “improvement” programmes [54]. While only 300 animals were registered in 2000, by 2018 the estimated population size was 2396 and had thus moved to being considered endangered rather than critically endangered [51].

As can be seen in Table 2, the proportion of the above animals which receive RDP support accounts for about half of the estimated population, with no clear links between the support levels (Table 1), participation rates, or the population sizes (Table 2).

**Table 2:** Estimated population of local breeds and share of the population included in the Rural Development Plan [51,55].

	Bela Krajina Pramenka		Drežnica Goat		Krškopolje Pig	
Year	Estimated Population	Share (%)	Estimated Population	Share (%)	Estimated Population	Share (%)
2000	250	-	550	-	300	-
2003	680	n.a.	600	n.a.	350	n.a.
2006	850	66.8	600	42.8	529	90.0
2009	880	50.1	600	40.3	658	72.1
2012	880	50.2	650	39.2	821	55.2
2015	930	44.2	660	49.5	1786	38.1
2018	1070	47.3	670	55.4	2396	46.1

### 2.2. Survey Approach

Drawing on the competitive tender approach applied under Payments for Agrobiodiversity Conservation Services (PACS) schemes [4,17], a stated preference survey of 301 Slovenian livestock farmers—divided equally between the three species—was carried out in the summer/autumn of 2015.

Farm-households were selected in the area of origin of the three local breeds, namely in Southeast Slovenia (Bela Krajina Pramenka sheep and Krškopolje pig) and the upper Soča valley (Drežnica goat). Through an initial visit to the farms by the research team, a participatory assessment, and interviews with key informants, preliminary information was collected. This included data regarding the development of the breed in the region, farmers’ experiences with the breed, trends and reasons for past population decline, as well as motives for keeping these breeds and associated costs. A pilot survey was also conducted among 30 randomly selected local breed farmers (10 per breed). After further consultations with a range of different policy, production, conservation and research stakeholders, the survey questionnaire was modified before full implementation. The final survey covered a total of 301 farm households. All farms engaged in breeding of the two critically endangered breeds (Bela Krajina Pramenka sheep and Drežnica goat) were included in the survey. A structured questionnaire was administered over 3 months and three experts from the local extension services were trained to conduct face-to-face interviews with the farmers. A closed-ended contingent valuation survey was used to determine the incentive payments required for farmers to be willing to maintain the local breeds in the future. Farmers were asked to express their willingness to accept with regard to two bid offers; while the first bid offer was randomly generated, the second one was related to the initial offer and the farmer’s response. Apart from contingent valuation, the survey included questions relating to the characteristics of the farm, socio-demographic aspects of the farm household and, of particular relevance to our study, farmers’ preferences and motives associated with maintaining the local breeds.

The objective of the survey was to assess farmers’ perceptions of the opportunity costs of maintaining those threatened breeds, with a view to assessing the cost-effectiveness of the current support scheme, which does not differentiate the level of payments between species, breeds and/or their threat status.

The survey covers approximately a quarter of Slovenia’s threatened local livestock breeds. Given that the three breeds differ in terms of species, threat status and potential for market valorisation, it is expected that wider implications for threatened AnGR management may be drawn from the results. Furthermore, the focus on a selected number of species and traits allows for robust research design and meaningful results to be generated from the use of the stated preference approach.

The survey covered existing local breed farmers, including 100% of all Bela Krajina Pramenka sheep and Drežnica goat farmers. It also included farmers of non-local breed—i.e., those with potentially favourable conditions for the introduction of the local breeds but not currently involved in rearing them.

Variables included in the analysis are briefly described in Table 3, while Table 4 provides descriptive statistics of the variables.

We used R 3.4.0 [56] to conduct the statistical analysis, namely logistic regression for the main model. The “dplyr” package [57] was used for data manipulation, “intReg” [58] for interval regression, “mfx” for marginal effects computation [59], and “ggplot2” [60] to produce the figures.

To identify other, non-monetary factors that affect farmers’ willingness to participate in support schemes, a logistic regression model was used. In this model, the dependent variable represents the farmer’s response to the proposed willingness to accept value. This is a binary variable that takes the value of 1 if the breeder accepts the proposed value and 0 otherwise. Explanatory variables classified as factors that affect the breeders’ willingness to participate in support schemes are presented in Table 4.

## 3. Results

Two-hundred and ninety-seven responses were obtained after removing those with missing values (4). Each respondent evaluated two bid offers (n = 594). The three local breeds which are the focus of this study comprised 21.7% of the total number of pigs, sheep and goats on the surveyed farms. This reflects both the fact that existing local breed farmers tend to also keep other breeds, as well as the fact that 62.1% of the farms included in the survey were not currently keeping local breed animals, even though they had potentially favourable production environments to do so. These farms were included in the survey as they could potentially switch to the production of local breeds and thus contribute to improving breed endangerment status.

The share of respondents engaged in rearing of local breeds ranged from 46% in the case of the Krškopolje pig, 29% in the case of the Bela Krajina Pramenka sheep and 23% in the case of the Drežnica goat. Differences in the number of surveyed farmers of local breeds primarily reflect differences in the total population and the number of farmers per breed. Farmers stated that they especially valued the Drežnica goat for its landscape conservation function (prevention of overgrowth), the Krškopolje pig for the quality of its traditional products and the Bela Krajina Pramenka sheep for both.

The average age of surveyed farm holders was 55 years, which is slightly below the national average (57 years). The majority of surveyed farmers were farming on a part-time basis. About two thirds of them (64.1%) have participated under past CAP agri-environmental schemes, but not all of them would wish to do so again due to what they considered to be burdensome administrative requirements and low payment levels under the previous programmes. This is despite the fact that average reported net annual household income amounted to less than EUR 15,000.

**Figure 1:**
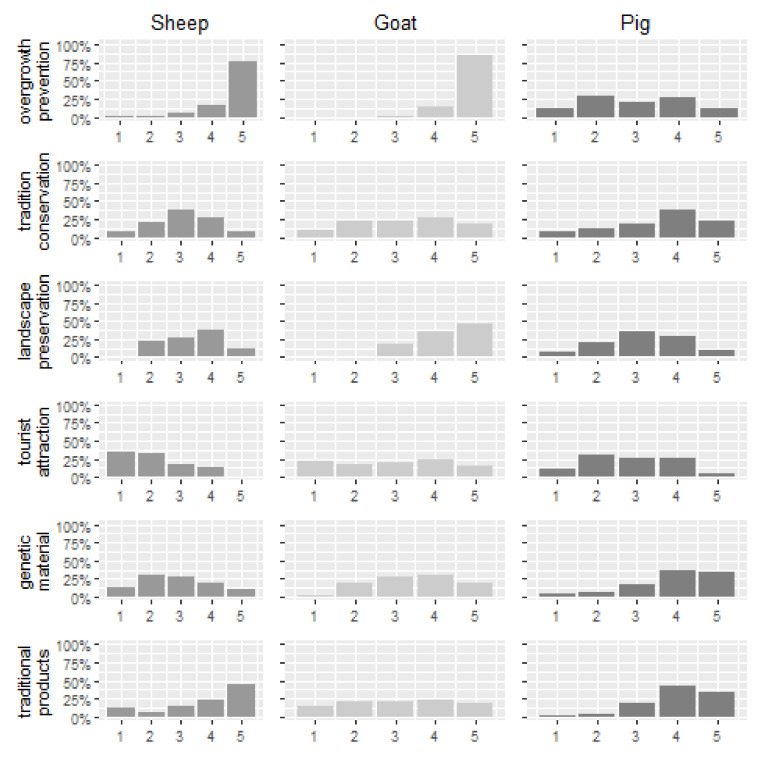
Farmer perceived environmental and social benefits of local breeds (Scale: 1—not important, 5—most important).

Farmers’ perceptions of environmental or social benefits arising from rearing local breeds by species are shown in Figure 1. While responses are broadly evenly distributed over the different categories, farmers most notably associate local goat and sheep breeds with the conservation of grassland use and traditional landscapes in marginal agricultural areas. Local pig and sheep breeds are also viewed positively with regard to the elaboration of traditional products, revealing their gastronomic potential.

Using interval regression analysis, average WTA amounts to EUR 21.26 for the conservation of local sheep and goat breeds and EUR 61.21 for local pig breeds (see Table 5).

In addition to WTA analysis, logistic regression was applied in order to check the consistency of WTA results and to reveal other relevant findings regarding farmers’ willingness to participate in the support scheme for local breeds. Results presented in Table 6 confirm that the amount offered for local sheep and pig breeds has a statistically significant positive impact on the decision to participate in support scheme (0.0597 and 0.0241, respectively). The relevant marginal effects are 0.0149 and 0.0060, which means that for each additional euro offered, the probability of accepting increases by 1.49 percentage points for sheep and by 0.60 percentage points for pigs.

## 4. Discussion

Given the fact that the survey was conducted at a time when the contracts for the 2007–2013 programming period were expiring (due to delays in the process of adopting the Rural Development Program 2014–2020, support for local breeds followed the RDP 2007–2013 rules) and some farms were still deciding whether to continue participating in the new scheme, it is interesting to compare the estimated WTA with the actual payment levels in force during the period 2007–2013 and the 2014–2020 period that was just starting at that time. The estimated WTA significantly exceeds the support levels available during the period 2007–2013, while approximating more closely those of the 2014–2020 implementation period at least for pig breeds (WTA 5% above RDP support levels). However, WTA estimates continued to be below actual payment levels for local sheep and goat breeds by 27%. This suggests that, particularly for the latter two local breeds, there is potential to improve the cost-effectiveness of the scheme.

The estimated *breed* coefficient (1.392) shows that farmers with a higher proportion of local breed animals in their herd are more likely to be willing to participate in support programmes. For each additional percent in the proportion of local breeds, the probability of the average farmer being willing to participate increases by 0.34 percentage points. This result has clear implications for policy. Farmers with breeding experience and whose production systems are adapted to the local breeds should be targeted as they are more likely to increase the number of animals included in the scheme. The finding is of particular relevance in the cases of local breeds with small populations [61].

The probability of participation in the support schemefor local breeds diminishes with the *age* of the farmer, though the relationship is not linear. The estimated *income* coefficient (−0.5167) means that farmers with higher levels of income are less likely to accept the amount offered. The probability of the average farmer accepting the amount offered decreases by 12.83 percentage points if farmer’s net annual household income surpasses EUR 15,000. Given the tendency of farmers’ income levels to increase over the long-term, this suggests that overall support costs may need to increase over time, although this could potentially be offset to some degree by specifically targeting younger and less wealthy farmers.

Farmers who had already participated in previous agri-environmental programmes (*AEP_12*) are less likely to be willing to participate in a future support scheme (−0.4515). The probability of the average farmer agreeing to participate decreases by 11.14 percentage points if farmers had participated in a previous programme; while increasing by 10.68 percentage points for those farmers participating in the current programme. A degree of dissatisfaction is thus observable regarding the implementation of previous agri-environmental measures. Three quarters of currently non-participating farmers cited their reasons for non-participation as being burdensome administrative requirements, restrictive compliance conditions or inadequate payment amounts. The challenge here is therefore associated with the redesigning of implementation arrangements in a way to better address the capabilities and needs of the breeders.

Results of the logistic regression (Table 6, marginal effects in Appendix A, Table A1) suggest that farmers’ perception of wider environmental and social benefits associated with local breeds may also affect their willingness to participate in related support schemes. One such benefit is overgrowth prevention, which is primarily associated with extensive use of grassland by grazing livestock. Farmers that perceive this benefit positively are found to be statistically significantly inclined towards participation in the support scheme (0.2289). In essence, the results confirm not only that farmers’ perceptions about the environmental and social benefits of the local breeds matter, but also increase their participation in related support schemes. Moreover, awareness and positive perception of environmental and social benefits of local breeds are a precondition for a successful (market- or policy-related) valorisation of these local breeds along the value chain [62].

**Figure 2:**
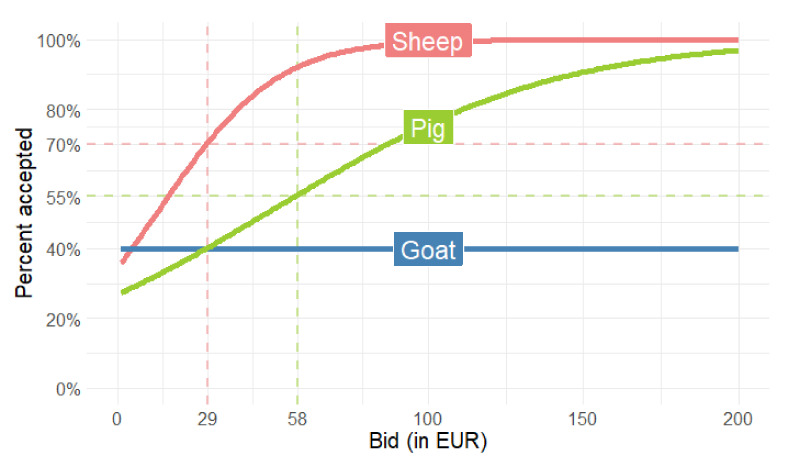
Farmers’ estimated participation in support schemes by payment level.

Figure 2 shows the predicted effect on farmer numbers of raising the payment amount to those levels that have applied under the 2014–2020 programme, i.e., EUR 29 for sheep and goats and EUR 58 for pigs. These values are indicated with the dashed line in Figure 2. Our model results suggest that the higher level of payments under the 2014–2020 programme should ensure the participation of 70% of sheep farmers and 55% of pig farmers. This is slightly above the latest (2018) population figures available (Table 2). While the model results reveal that sheep and pig farmers’ decisions to participate in support schemes are responsive to the payment level, this is not the case with the farmers engaging in the breeding of the local goat breed, whose vulnerability status is the least favourable of the three. Given the fact that the population of the goat local breed has been persistently low for the whole observation period (see Table 2), it appears that the current policy effort, including a dedicated agri-environomental scheme does not bring positive results. A long-term sustenance of the breed at risk such as in this particular case, is not achievable without an increase in the pool of breeding animals. Among the strategies to achieve this goal, FAO [49] recommends various options, which have not yet been fully exploited in this particular case: from community-based in situ conservation, establishment of breeders’ association, undertaking centralized ex situ conservation on institutional farms (alone or in combination with farmers’ herds) and innovative product marketing.

The importance of the latter strategy is supported by the recent favourable population dynamics of the Krškopolje pig population, which coincides with the growing demand for traditional meat products on the local market [63]. Positive experience encourages further action to improve the attractiveness of local breeds through the improved market visibility of traditional products involving local breeds [64]. Such actions could include establishing certification schemes and promotion of niche product markets, setting-up of producer groups, or facilitating cooperation in the development of innovative products and services in agriculture [65].

## 5. Conclusions

The paper contributes to empirical studies related to incentives for agrobiodiversity conservation services by extending such research to the area of animal genetic resources. We examine the support scheme for local breeds in Slovenia, which has been implemented within the context of the national agri-environmental programme. The measure has been based on fixed payments per LU. Participation of farmers in the scheme remained relatively constant over time, irrespective of the level of payments, which varied significantly from one program period to the other. This result leads to our first policy recommendation, suggesting that cost-effectiveness of AnGR conservation support schemes could be improved through the use of differentiated payment levels that more accurately reflect farmers’ true opportunity costs.

More attention should also be paid to the implementation arrangements, as was also noted in the Romanian context [18], which is revealed to be an important cause for farmer dissatisfaction with the current scheme. Such dissatisfaction primarily results from the fact that obligations are either difficult to accommodate in practice or are time-consuming and do not generate value for their operations. In our view, burdensome administrative requirements are a valid point for our second policy recommendation. Measures to streamline administrative requirements are required to encourage broader farmer participation and may also be facilitated through a transition to a results-based principle of support payments.

Another aspect of the study that deserves further exploration deals with the need for complementary strategies to support the sustainable conservation of animal genetic resources, with regard to enhancing both their public and private good values (as per Matin-Collado et al. [29] and Zander et al. [30]). Market valorisation of local breeds is one such strategy. The distinctive characteristics of local breeds could serve as an attribute for adding value [66]. Alternatively, agricultural systems providing ecosystem services could also be provided through voluntary action [67]. In the particular case of the conservation of animal genetic resources, such incentives might include the public-private conservation schemes, supported by local communities.

A precondition for the viability of the above approaches is associated with societal (or market, when a monetary value can be assigned) demand. Such an outcome requires a long-term development process along the value chain [62]. Private sector approaches may include price premiums based on labeling or branding schemes [68], often based on some form of quality, social or environmental certification [69]. As a note of caution, though, Narloch et al. [4] warn that niche product market development may raise the financial profitability only of those characteristics that closely match consumers’ current tastes and preferences. Furthermore, this study has shown that farmer awareness of the wider environmental and societal benefits of local breeds increases their participation in the support scheme. Awareness is also a necessary step to raise the appreciation of these benefits by society at large. In the same vein, our third policy recommendation highlights the importance of measures that raise awareness of the wider environmental and societal benefits of local breeds. This is a precondition for ensuring higher levels of farmer participation, as well as contributing to higher levels of public appreciation that can potentially be translated into long-term sustainable value chain development, and the sustainable conservation of animal genetic resources in the long run.

## Figures and Tables

**Table 1:** Overview of monetary incentives for rearing the local breeds in Slovenia from 2004 to 2020 *.

	Payments in EUR Per Unit		
Period	Sheep/Goat (Head)	Pig (Head)	Livestock Unit (1 LU = 500 kg)
2004–2006	18.00	48.00	/ **
2007–2013	13.41	26.81	89.38
2014–2020	29.04	58.08	193.62

* Payment levels are reported only for species and categories within the scope of this study. Livestock Unit equivalents are as follows: local sheep and goats (both 0.15 LU) and pigs (0.3 LU). ** in 2004–2006, the system was based on fixed payments per animal, differentiated by species.

**Table 3:** WTA model variables.

Variable	Description	Unit
Species	Species of animals	1 = sheep 2 = goat 3 = pig
Breed	Share of local breeds in total population	%
Age	Farmer’s age	Years
Income	Net annual household income	0 = lower than EUR 15,000 1 = higher than EUR 15,000
Employment	Farmer’s employment status	1 = agricultural employment 2 = non-agricultural employment 3 = unemployed 4 = retired
AEP_12	Participation in past agri-environmental programme	0 = no; 1 = yes
AEP_3	Willingness to participate in future agri -environmental programme	0 = no; 1 = yes
Accept	Acceptance of the proposed support level	0 = not accept 1 = accept
Bid	Bid amount for local breeds	EUR
	Environmental & social benefits of local breeds:	
overgrowth	prevention of overgrowth	
tradition	conservation of tradition	Scoring of importance:
landscape	conservation of landscape	
Tourism	attraction for tourists	1-not important
gen_mat	source of genetic material	to
product	traditional products	5-most important

**Table 4:** Descriptive statistics.

Variable	N	Mean	SD
Species	297	1.997	0.818
Breed	297	0.217	0.313
Age	297	55.189	13.627
Income	297	0.412	0.493
Employment	297	2.340	1.289
AEP_12	297	0.690	0.463
AEP_3	297	0.439	0.497
Accept	297	0.519	0.500
Overgrowth	297	4.151	1.195
Tradition	297	3.291	1.172
Landscape	297	3.572	1.075
Tourism	297	2.602	1.238
gen_mat	297	3.371	1.200
Product	297	3.656	1.288

AEP_12—Participation in past agri-environmental programme; AEP_3—Willingness to participate in future agri -environmental programme.

**Table 5:** WTA interval regression estimation results.

Dependent Variable	WTA		
Explanatory Variable	b_i_	t	*p* (t)
Constant	21.2621	15.037	0.000 ***
Goat	−0.4284	−0.154	0.878
Pig	39.9541	23.797	0.000 ***
N	226		
Df	222		
Sigma	9.9579	21.375	0.000 ***

Significance levels: *p* < 0.1; * *p* < 0.5; ** *p* < 0.01; *** *p* < 0.001.

**Table 6:** WTA logistic regression estimation results.

	WTA		
Explanatory Variable	b_i_	z	*p* (z)
Constant	0.6504	0.45	0.652
breed	1.3921	3.91	0.000 ***
age	−0.1281	−2.78	0.005 **
I (age*2)	0.0010	2.31	0.021 *
Income	−0.5167	−1.95	0.051
non−agricultural employment	0.4873	1.81	0.070
unemployed	−0.5401	−0.820	0.412
retired	0.5811	1.99	0.046 *
AEP_12	−0.4515	−1.76	0.079
AEP_3	0.4306	1.80	0.072
overgrowth	0.2289	2.06	0.039 *
tradition	0.2550	2.57	0.010 *
landscape	−0.0583	−0.53	0.597
tourism	−0.0844	−0.98	0.326
gen_mat	0.0071	0.07	0.942
product	0.2078	2.27	0.023 *
bid:sheep	0.0597	4.17	0.000 ***
bid:goat	−0.0020	−0.14	0.885
bid:pig	0.0241	4.45	0.000 ***
N	594		
AIC	713.02		

Significance levels: *p* < 0.1; * *p* < 0.5; ** *p* < 0.01; *** *p* < 0.001.

## Data Availability

The data used herein may be made available upon reasonable requests to the Biotechnical Faculty, Department for Animal Science by contacting authors.

## Appendix A

**Table A1:** WTA logistic regression estimation results, marginal effects.

Dependent Variable	WTA		
Explanatory Variable	dF/dx	z	*p* (z)
breed	0.3471	3.92	0.000 ***
age	−0.0319	−2.78	0.005 **
I (age*2)	0.0002	2.31	0.021 *
income	−0.1283	−1.97	0.049 *
non−agricultural employment	0.1198	1.85	0.064
unemployed	−0.1335	−0.85	0.398
retired	0.1429	2.04	0.041 *
AEP_12	−0.1114	−1.79	0.074
AEP_3	0.1068	1.82	0.069
overgrowth	0.0571	2.06	0.039 *
tradition	0.0636	2.57	0.010 *
landscape	−0.0145	−0.53	0.597
tourism	−0.0210	−0.98	0.326
gen_mat	0.0018	0.07	0.942
product	0.0518	2.27	0.023 *
bid:sheep	0.0149	4.17	0.000 ***
bid:goat	−0.0005	−0.15	0.885
bid:pig	0.0060	4.46	0.000 ***

Significance levels: *p* < 0.1; * *p* < 0.5; ** *p* < 0.01; *** *p* < 0.001.

## Appendix B

**Table A2:** Population trends (number of animals) and incentive payments for the three analysed breeds, for period 2000–2018.

Breed	Endangerment Status in 2018		2000	2003	2006	2009	2012	2015	2018
**Belokranjska pramenka sheep**	Critically endangered	population	250	680	850	880	880	930	1070
		incentive payments €/head	/	18		13		29	
		% participation	n.a.	n.a.	66.8	50.1	50.2	44.2	47.33
**Drežnica goat**	Critically endangered	population	550	600	600	600	650	660	670
		incentive payments €/head	/	18		13		29	
		% participation	n.a.	n.a.	42.8	40.3	39.2	49.5	55.37
**Krškopolje pig**	Endangered	population	300	350	529	658	821	1786	2396
		incentive payments €/head	/	48		27		58	
		% participation	n.a.	n.a.	90.0	72.1	55.2	38.1	46.10

## Appendix C

**Table A3:** Population trends (number of animals) of Slovenian local breeds for the period 2000–2018.

Breed/Year	2000	2003	2006	2009	2012	2015	2018	Threat Status in 2018
Lipizzan horse	600	630	1000	800	1.150	1.250	1.267	critical
Slovenian cold-blooded horse	n.a.	1.120	2.200	3.000	3.420	3.000	3.100	critical
Posavje horse	n.a.	700	630	900	1.560	1.700	1.880	critical
Cika cattle	400	686	1.350	2.159	2.858	3.784	4.905	endangered
Krško Polje pig	300	350	529	658	821	1.786	2.396	endangered
Jezersko—Solčava sheep	19.000	19.200	17.000	17.200	17.200	17.000	15.000	vulnerable
Bovec sheep	3.500	3.600	3.600	3.500	3.500	3.300	3.800	critical
Bela Krajina Pramenka sheep	250	680	850	880	880	930	1.070	critical
Istrian pramenka sheep	600	1.200	1.100	1.150	1.150	1.020	1.100	critical
Drežnica goat	550	600	600	600	650	660	754	critical
Styrian hen	1.000	1.200	1.000	1.200	1.800	1.700	1.600	endangered
Carniolan honey bee (families, in thousands) *9	162	157	171	143	150	150	180	/

Significance levels: *p* < 0.1; * *p* < 0.5; ** *p* < 0.01; *** *p* < 0.001.
